# VeloxChem: GPU-Accelerated Fock Matrix Construction Enabling Complex Polarization Propagator Simulations of Circular Dichroism Spectra of G-Quadruplexes

**DOI:** 10.1021/acs.jpca.4c07510

**Published:** 2024-12-31

**Authors:** Xin Li, Mathieu Linares, Patrick Norman

**Affiliations:** † PDC Center for High Performance Computing, KTH Royal Institute of Technology, SE-100 44 Stockholm, Sweden; ‡ Division of Theoretical Chemistry and Biology, School of Engineering Sciences in Chemistry, Biotechnology and Health, KTH Royal Institute of Technology, SE-100 44 Stockholm, Sweden

## Abstract

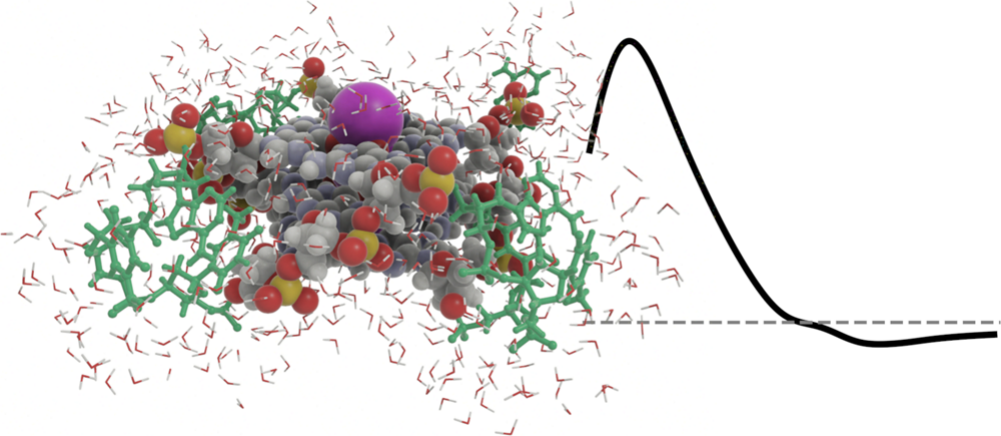

An automatic code generated C++/HIP/CUDA implementation of the (auxiliary) Fock, or Kohn–Sham, matrix construction for execution in GPU-accelerated hardware environments is presented. The module is developed as part of the quantum chemistry software package VeloxChem, employing localized Gaussian atomic orbitals. The performance and scaling characteristics are analyzed in view of the specific requirements for self-consistent field optimization and response theory calculations. As an example, the electronic circular dichroism spectrum of a G-quadruplex is calculated at the level of time-dependent density functional theory in conjunction with the range-separated CAM-B3LYP exchange–correlation functional. Computational issues due to the high-density of states following the adoption of large-scale model systems are here bypassed with use of the complex polarization propagator approach. The origin of the negative Cotton effect in the long-wavelength onset of the experimental spectrum is elucidated in large-scale modeling and shown to be associated with the TTA nucleobase linkers in the G-quadruplex.

## Introduction

In life sciences and materials chemistry, molecular systems are pivotal, and studies of their microscopic structures and chemical and physical processes are routinely performed to gain an understanding of the macroscopic function, performance, and properties; these studies can be experimental, theoretical, or a combination of the two, and arguably, a strong point of the latter discipline is the possibility to relate molecular and electronic structures to properties and observables. Today, there is a plethora of spectroscopies in use for the characterization of molecular materials and systems^1^ and an associated rich variety of theoretical methods to perform simulations of these, none more impactful than those based on time-dependent density functional theory (DFT). The notion of chemically intact subsystems and localized electronic transitions has further led to the development of physically well-motivated polarizable embedding techniques that has enabled mixed quantum–mechanical/molecular–mechanical (QM/MM) spectroscopy simulations involving complex molecular systems.^2^ However, the size of the QM region can ultimately not be made smaller than the spatially most widespread transition density in the spectral bands of interest.

In cases when the nature of the system and electronic transitions are such that a large-scale QM region is called for, then one, in addition, typically faces the challenge of high densities of states, even if the gap energy itself is sizable. This is computationally prohibitive for the bottom-up approaches used to solve the generalized eigenvalue equation in time-dependent DFT. One way to circumvent this issue is to adopt the complex polarization propagator (CPP) formulation of response theory where the account made for the finite lifetimes of the excited states makes response functions physically sound also in resonance regions of the spectrum with real and imaginary parts being related by Kramers–Kronig relations.^3−5^ The underlying excited states are not resolved in the calculation, which instead focuses on determining the response functions pertinent to the spectroscopy at hand for a discretized frequency window enclosing the sought for spectral bands. With a solution of the associated set of response equations made in parallel, the convergence can be highly accelerated.^6^ The CPP theory has been implemented in several quantum chemical programs and thereby for most standard electronic theory methods.^5^ In this work, we will employ the DFT implementation of real and complex linear response theory made in the VeloxChem software^7^ and introduce a GPU-accelerated construction of the regular Fock (or Kohn–Sham) matrix needed for the self-consistent field (SCF) optimization of the reference state as well as the auxiliary Fock matrices needed in the response part of the calculation.

In DFT calculations, the computational complexity mainly arises from the need to evaluate electron repulsion integrals (ERIs) and exchange–correlation (XC) contributions.^8,9^ The past decades have witnessed the development of hardware and algorithms that enabled routine DFT studies of molecular systems consisting of up to several hundreds of atoms^10,11^; however, regular DFT and linear response time-dependent DFT calculations of larger systems with some few thousands of atoms remain challenging, especially for multichromophoric systems as due to the need to solve for many closely lying excited states. In recent years, along with the rapid development of heterogeneous computing and hardware accelerators such as GPUs, significant progress has been made in enabling DFT calculations on GPUs.^12−26^ These efforts focus on redesigning the algorithms for integral evaluation, XC integration, and Fock matrix formation such that the highly parallel architecture of GPUs can be efficiently utilized. Moreover, use of dynamic precision,^12,27^ automated code generation,^28,29^ evaluation of ERIs with higher angular momenta,^30−32^ and time-dependent DFT implementations (within the Tamm–Dancoff approximation)^33,34^ on GPUs have also been reported. The use of GPUs in DFT calculations has therefore been greatly promoted. However, to the best of our knowledge, a GPU implementation of the CPP formulation of linear response theory has not been reported before, yet being particularly well suited for studying spectra of large and complex multichromophoric systems.

Electronic circular dichroism (CD) spectroscopy has emerged as a powerful tool for studying the conformational properties of DNA. The ability to accurately calculate and predict CD spectra for DNA structures is crucial for interpreting experimental data and gaining deeper insights into DNA conformations and dynamics. One of the fundamental challenges in calculating CD spectra for DNA lies in the complexity of the molecular system. As highlighted by Kypr et al.,^35^ DNA can adopt a variety of conformations, including the B-family of structures, A-form, Z-form, guanine quadruplexes, cytosine quadruplexes, and triplexes. Each of these structures exhibits distinct CD spectral signatures, making the accurate calculation of CD spectra a nontrivial task. In addition to the nucleobases, sugars and phosphate groups can also play a role and need to be considered for a proper evaluation of the CD signal.

Over the years, various approaches have been developed to calculate CD spectra for DNA, ranging from empirical methods to advanced quantum mechanical calculations. In 2015, Monari et al. proposed a multiscale approach to calculate the CD spectra of B-DNA.^36^ Their method combined quantum mechanical calculations of individual nucleobases with snapshots selected from molecular dynamics simulations. Excited states of individual base pairs were obtained using time-dependent DFT in a QM/MM setting and used to build a Frenkel exciton model. This approach allowed them to account for the effects of structural fluctuations on the exciton coupling between DNA bases and provided a more accurate representation of the coupling compared with the dipole approximation. This approach was then successfully used to investigate the CD spectra of DNA G-quadruplexes.^37^ The authors demonstrated that their method accurately reproduces the experimental CD spectra for various G-quadruplex topologies, highlighting the significant influence of G-tetrad stacking interactions on spectral features. The approach successfully distinguishes between parallel and antiparallel G-quadruplex arrangements while claiming that loop nucleotides have a minimal impact on the overall spectra. Aside from the excitonic model, several approaches have been used to calculate CD spectra of DNA by finding the smallest minimal model to reproduce experimental results. We have for instance shown that a dimer of base-pair representative of a double strand of 20 base pairs can be used to reproduce accurately the experimental spectra.^38^ We then successfully applied this approach to reproduce the decrease in intensity observed in nucleosomal DNA upon wrapping around the nucleosome.^39^ The choice of this small model was obviously guided by the limitation in the system size that could be treated with the software available at that time. Given the general performance of VeloxChem on modern hardware and the GPU implementation presented here, we make an attempt to push the boundaries of DNA modeling and revisit the case of the G-quadruplex.

## Implementation

We present a GPU implementation of linear response theory in the Kohn–Sham DFT approximation with an adoption of localized Gaussian atomic orbitals; it encompasses the generalized eigenvalue equation as well as the complex polarization propagator. In the iterative linear response equation solvers, we adopt the use of symmetric and antisymmetric trial vectors as introduced by Kauczor et al.^6,40^ for the reduced space algorithms. The key compute intensive operation is the construction of (auxiliary) Fock matrices from density matrices that are either symmetric or antisymmetric depending on the symmetry of the trial vectors. This Fock matrix construction, in turn, has two separate parts of significance to the computational cost, namely, the contraction or the ERI tensor with said density matrices and the numerical DFT kernel integration, both of which are carried out with use of GPUs in the present work.

### Evaluation of Electron Repulsion Integrals

The ERIs are evaluated using the Obara–Saika scheme.^41^ To facilitate integral calculations on GPUs, we avoid the contraction step on the device and instead compute all ERIs in the primitive basis. Moreover, we chose to evaluate the Cartesian components of the primitive functions separately such that the ERIs involving different Cartesian components can be performed on separate threads. For example, a primitive [sp|sp] integral is split into nine ERI evaluations that can be carried out independently. The code for computing the ERIs is autogenerated by explicitly “unrolling” all the Obara—Saika vertical and horizontal recurrence relations, and each ERI can thus be computed from scratch without using any intermediate results from other ERIs. The final expressions for ERIs involving *s*- and *p*-functions are similar to the explicit formulas presented by Baxter and Cook,^42^ and we have generated ERI expressions up to *d*-functions.

#### Coulomb and Exchange Contributions

The Fock matrix is formed by contracting the ERIs with the density matrix. At the Hartree–Fock and hybrid DFT levels of theory, the two-electron part of the Fock matrix consists of two contributions with different contracting patterns, namely, the Coulomb and the exchange contributions, which are denoted as **J** and **K**, respectively. To reduce the computational cost of the Fock matrix formation, which formally scales as *O*(*N*^4^) with *N* being the number of basis functions, it is essential to make use of efficient screening of integrals based on the Schwarz upper bound^43^
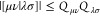
1where  and μ and ν denote Cartesian components of the primitive basis functions such as s, p_*x*_, d_*xy*_, etc.

In our implementation, we followed the approach proposed by Ufimtsev and Martinez,^15^ where the **J** and **K** matrices are formed separately to maximize data locality and to avoid communication between thread blocks. In this approach, the [μν| and |λσ] pairs are presorted based on the values of *Q*_μν_ and *Q*_λσ_|*D*_λσ_|, respectively, as to ensure efficient screening of ERIs in the calculation of the **J** matrix
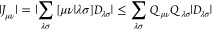
2This allows for a full exploitation of the *O*(*N*^2^) scaling of the Coulomb contribution. For the **K** matrix, the presorting is instead carried out for subgroups of the [μλ| and |νσ] pairs where the first primitive indices correspond to the subscript of the **K** matrix elements such that a thread block can scan over relevant pairs to form a specific **K** matrix element (see ref (15) for details). To take advantage of the *O*(*N*) scaling of the exchange contribution, it is important to make use of the sparsity of the density matrix in the screeching of integrals. In our implementation, instead of using the “guard” parameter as described in the work of Ufimtsev and Martinez,^15^ we adopted the preselective screening (preLinK) approach proposed by Kussmann and Ochsenfeld,^18^ where the upper bound of the exchange matrix element is written as
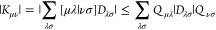
3and can be conveniently computed by matrix multiplication.

To make efficient use of GPUs, different kernels need to be implemented to handle different combinations of angular momenta in the ERIs. For the **J** matrix, where the [μν|λσ] integrals need to be evaluated, we need ss, sp, pp, etc. for the μν indices and same goes for the λσ indices. This results in 9 Coulomb kernels for up to p-functions and in our case 36 for up to d-functions. For the **K** matrix, where the [μλ|νσ] integrals need to be evaluated, we need ss, sp, pp, etc. for the μν indices, while for the λσ indices we need to take into account all combinations including ss, sp, ps, pp, etc. This leads to 12 exchange kernels for up to p-functions and in our case 54 for up to d-functions. Note that we only take into account the upper triangular combinations for the μν indices when evaluating *J*_μν_ and *K*_μν_, since we only need to deal with symmetric and antisymmetric density matrices.^40^ Also, for antisymmetric density matrices, we skip the formation of the **J** matrix. After the **J** and **K** matrices are formed in the primitive basis, the transformation to contracted basis is carried out on the node CPU.

### Exchange–Correlation Numerical Integration

Numerical integration of the exchange–correlation (XC) contribution to the Fock matrix can be efficiently carried out on GPUs. In this work, we follow the approach proposed by Ochsenfeld and coworkers,^26^ where the evaluation of densities on the grid points and XC contribution to the Fock matrix is done by matrix multiplication which can make efficient use of the GPUs. Moreover, the *O*(*N*^3^) computational cost in XC integration, where *N* is the system size, can be greatly reduced by integrating over even-sized grid batches such that the computational cost becomes asymptotically linear due to efficient screening of basis functions.^26^ The key to such an efficient implementation is to make sure that the DFT grid points of the whole system are divided as evenly as possible into batches of spatially adjacent grid points. In this work, this is realized by repeatedly bisecting the box of grid points into smaller and smaller boxes until the number of grid points in each box is below a given threshold. The bisection of boxes is done as evenly as possible (in terms of the number of grid points) such that the final boxes of grid points have almost constant computational cost on average. Through matrix multiplication, the majority of the computation steps of XC numerical integration are done on GPUs, while the evaluation of functional derivatives, for which we use the Libxc library,^44^ is done on the node CPU.

### Performance and Scaling

In spectroscopy simulations using time-dependent DFT, calculations have two distinct parts where the first involves the SCF optimization of the electron density of the unperturbed reference state and the second involves the application of response theory to determine the time-dependent oscillations in the electron density due to the external electromagnetic fields. The overarching goal of our software development is to minimize the total wall time required to produce the final spectra of realistic model systems. For biochemical systems, in particular, that requires attention to be paid to both the environment (often aqueous solutions and/or protein embeddings) and the effects of molecular dynamics (typically handled by configuration sampling). The former of these two aspects is here taken into account with use of QM/MM modeling, whereas the latter aspect is not addressed. Configuration space sampling is, however, embarrassingly parallel in nature and can therefore be left out of the discussion of computational performance and scaling. From a physical standpoint, it is also well motivated to leave out this issue in the case of our specific application, as the G-quadruplex has a very low degree of conformational flexibility.

The SCF optimization involves the construction of a single Fock matrix at a time with a typical repetition of a few tens of times. In contrast, the response calculation involves the need to construct multiple Fock matrices with a number of matrices that can vary quite strongly over the course of the iterations in the iterative linear response equation solver. In the present work, we have not pursued any attempts to leverage the fact that the construction of multiple Fock matrices can be made in parallel in the sense that the set of ERIs can be computed only once but in conjunction with separate density matrix contractions and distributions, albeit with a penalty in the form of screening efficiency. The total number of Fock matrices constructed in the response part of the calculation typically amounts to several thousands in a spectrum simulation for a medium- or large-scale system, and the associated computational efforts therefore dwarf those made in the SCF optimization.

With increasing system size, however, the SCF optimization will eventually start to become a computational bottleneck due to the -scaling of the diagonalization of the Fock matrix and which is performed in every iteration of the SCF driver to obtain molecular orbitals (MOs) and form an updated density matrix. We carry out this matrix diagonalization on a single GPU node with use of the MAGMA library^45^ (version 2.8.0) for linear algebra on AMD GPUs using the HIP programming model.^46^

In Table 1, we present wall clock timings for all parts of the calculations that are significant to the overall performance, and we do so for the small, medium, and large models of the G-quadruplex that will be presented below in detail. In this range of system sizes (up to some 12,000 basis functions), the results amply demonstrate that the contraction of ERIs with density matrices by a wide margin represents the most costly part of the calculation. The numerical integration of the XC kernel benefits from a very efficient screening, and as made clear from the results, the ratio of computational efforts between the ERI and XC parts monotonically increases with system size. The same is not true for Fock matrix diagonalization in the SCF iterations, but even for the largest model with 11,934 basis functions, the associated wall time reaches a mere 11.3 s. The present implementation strategy, hardware, and math library make it affordable to treat systems up to and somewhat beyond 30,000 basis functions in the SCF optimization.

**Table 1:** Wall Time (in Seconds) Spent in ERIs, XC Kernel Integration, and Fock Matrix Diagonalization in One SCF Iteration of the Series of Three Model Systems, Measured on Four GPU Nodes or 16 MI250X GPUs (Each with Two GCDs).

model	*N*_basis_	*t*_ERI_	*t*_XC_	*t*_diag_
small	2,436	12.9	0.4	0.4
medium	3,972	31.4	0.7	1.5
large	11,934	340.7	5.1	11.3

**Figure 1:**
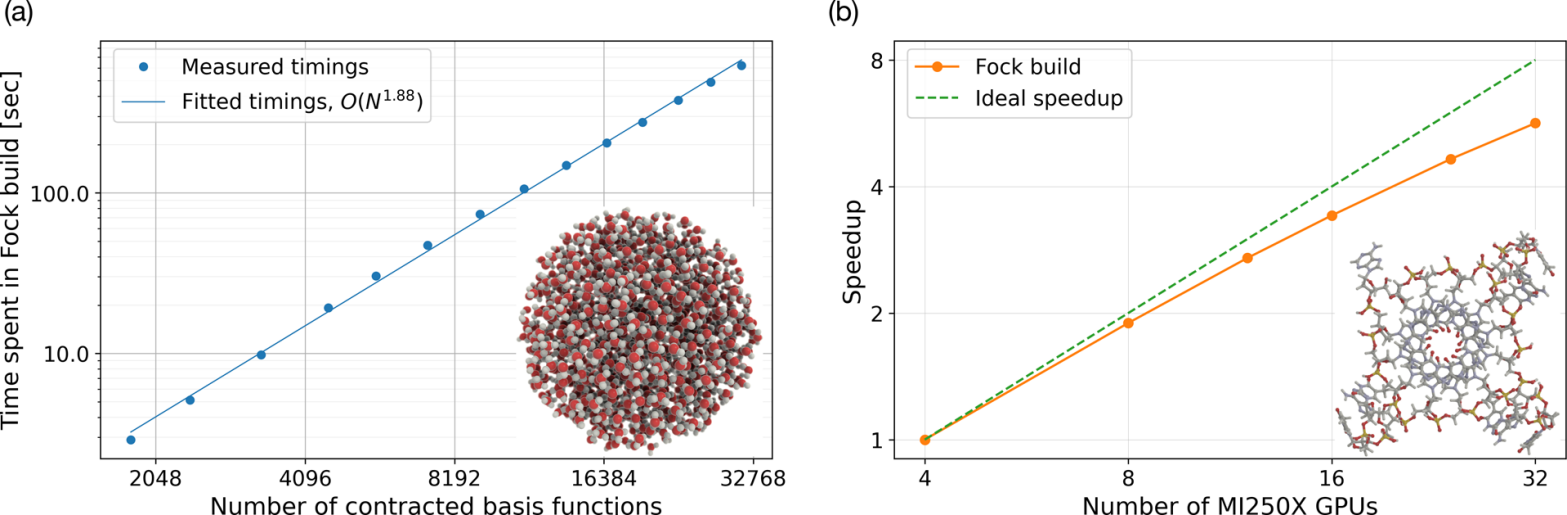
Two separate scaling aspects of the GPU implementation of the ERI-part of the Fock matrix construction: (a) system size scaling illustrated by the wall times (in seconds) on a single GPU node obtained for spherical water clusters of varying sizes (the inset shows the largest cluster). (b) Strong scaling with respect to the number of GPUs (each with two GCDs), illustrated here by a G-quadruplex including all nucleotides where the phosphate group has been neutralized by adding a hydrogen. Potassium ions and the environment have been removed. In both cases, the def2-SVP basis set was adopted.

Having established that the ERI part of the Fock matrix construction dominates the computational cost for system sizes of interest for running on GPU-accelerated clusters, we turn to the observed scaling behaviors of our software implementation. In Figure 1a, we present the scaling with respect to the system size by keeping the resource constant (one node with four GPUs) while increasing the size of the spherical water droplet. This is a dense 3D system, and our observations should thus not be contaminated by artificial sparsity and favorable screening that can be found in systems of more 1D or 2D character. The ratio between “heavy” and hydrogen atoms is here 1:2, which is quite representative albeit somewhat lower than the typical application where, e.g., aromatic rings would push the ratio upward. This example does employ d-functions in the basis set for oxygen, and the associated integral code kernels are thus here brought into action. The reason why this deserves to be mentioned is that these kernels are much more extended (number of source code lines) than those involving only lower angular momenta and thereby more difficult to launch efficiently on GPUs. This example does, however, not employ any diffuse basis functions that would hamper screening and push the true scaling of the implementation to be observed for larger system sizes. We argue, however, that DFT linear response properties for large-scale 3D systems often are reasonably well described with a basis set such as def2-SVP such that this example becomes relevant for real case studies. The observed scaling with respect to system size (or number of basis functions) is *N*^1.88^, which is a testament of the quadratic scaling of the **J** matrix mixed with the incipient linear scaling of the **K** matrix.

In Figure 1b, we present the strong scaling of our implementation. The test presented in this figure is carried out for a system that is intermediate in size to the medium and large models of the G-quadruplex; it contains the nucleotides but not the potassium ions or the environment. In this test, we can employ up to eight nodes (or equivalently 32 GPUs, each with two graphics compute dies, GCDs) and maintain an efficiency in parallelization that exceeds 70%.

This strong scaling aspect is of great importance for spectroscopy simulations (more so than energy calculations) simply due to the very large number of Fock matrices that need to be determined, and arguably, it makes sense to view the number of Fock matrices produced per hour as a key performance indicator (KPI) of the code. From Table 1, we see that this KPI for the large model system and using four nodes is equal to 10.4 h^–1^—as given by 3600 s/h divided by (340.7 + 5.1) s. For energy calculations, this number is more than adequate, but for spectrum simulations, it is just barely sufficient for practical work as it will take several hundreds of wall time hours to produce a spectrum. This KPI number is determined from data taken from the SCF optimization where the density matrices are always symmetric. In the response part (that dominates the computational cost), there is a mix of symmetric and antisymmetric auxiliary density matrices, and for the latter ones, there is no Coulomb contribution. Taking the average of 105 Fock builds in one prototypical iteration of the linear response equation solver, we obtain a KPI of 14.1 h^–1^ with the use of four nodes. On eight nodes, the KPI number reaches a value of 20.7 h^–1^ when averaged over 105 Fock builds, and this number can be pushed a bit further with use of more nodes simply because the strong scaling performance should benefit from the system being larger than that used in Figure 1b. With the use of 12 and 16 nodes, we reach KPI results of 24.3 and 26.3 h^–1^, respectively.

## Application

### Computational Details

The molecular structures of the model systems were based on the parallel stranded quadruplex of d(G4) stabilized by three potassium ions with ID 1KF1^47^ in the protein data bank (PDB). The full sequence of this DNA strand is included in our simulation (largest model system) and reads A*GGG*TTA*GGG*TTA*GGG*TTA*GGG*, where the guanine nucleotides forming the three layers of the G-quadruplex are written in italic.

This system was immersed in a box of TIP3P^48^ water of dimension 15 × 15 × 15 nm^3^, and 18 sodium ions were added to neutralize the overall charge of the box. The AMBER99SB-ILDN force field^49^ was used for DNA and ions. A short MD simulation of 5 ns was performed in the *NPT* ensemble to optimize the density of the box. The velocity rescaling weak coupling scheme^50^ with a coupling constant of τ = 0.2 ps was used to maintain the reference temperature of 300 K. The pressure was maintained at 1 atm using the Berendsen barostat^51^ with a coupling constant of 1 ps. Then, an *NVT* simulation of 20 ns was performed with the same thermostat as that previously described. In both the *NVT* and *NPT* simulations, the particle mesh Ewald (PME) approach^52^ was used to model electrostatic interactions with a long-range cutoff of 1.5 nm. Lennard–Jones interactions were also modeled with the same cutoff of 1.5 nm. The time step for the MD simulations was 2 fs. The backbone of DNA was restricted using a position constraint of 1000 kJ·mol^–1^·nm^–2^. During all MD simulations, bonds involving hydrogen atoms were constrained using the LINCS algorithm.^53^ Finally, the final snapshot structure was optimized with respect to the adopted molecular mechanics (MM) force field, from which three model systems were constructed as described in detail in the subsequent section. These preparatory calculations were carried out with use of the Gromacs program.^54^

In the spectrum simulations, the def2-SVP^55^ basis set was employed for all atoms belonging to the G-quadruplex, including the potassium ions. The largest model system has in addition to this core region also an embedding region consisting of (i) a QM solvation shell for which the STO-3G^56^ basis set was employed for water molecules and sodium ions and (ii) an MM solvation shell with point charges of −0.6690 and +0.3345 representing the oxygen and hydrogen atoms in water and +1 representing sodium atoms. The range-separated CAM-B3LYP exchange–correlation functional^57^ was adopted throughout the present work with the original parameter settings of α = 0.19, β = 0.46, and ω = 0.33. In CPP calculations, the program default damping parameter of γ = 0.1240 eV (or 1000 cm^–1^) was adopted; in calculations using the generalized eigenvalue solver, the corresponding half-width at half-maximum (HWHM) parameter was used for the spectral broadening of the rotatory strengths.

All QM/MM simulations were conducted with a locally modified version of the VeloxChem program^7^ equipped with alternate GPU modules for the integral evaluations and XC kernel integrations required for the Fock matrix constructions in the SCF and response parts of the calculations. These calculations were carried out on the GPU partition of either the Dardel cluster at the PDC Center for High Performance Computing or the LUMI cluster at the CSC-IT Center for Science. In both cases, the node configuration comprised four AMD Instinct MI250X GPU chips, each with two GPU devices known as graphics compute dies (GCDs). A locally modified version of the VIAMD software^58^ was used to visualize the molecular and electronic structures of the three model systems.

### Building Model Systems

**Figure 2:**
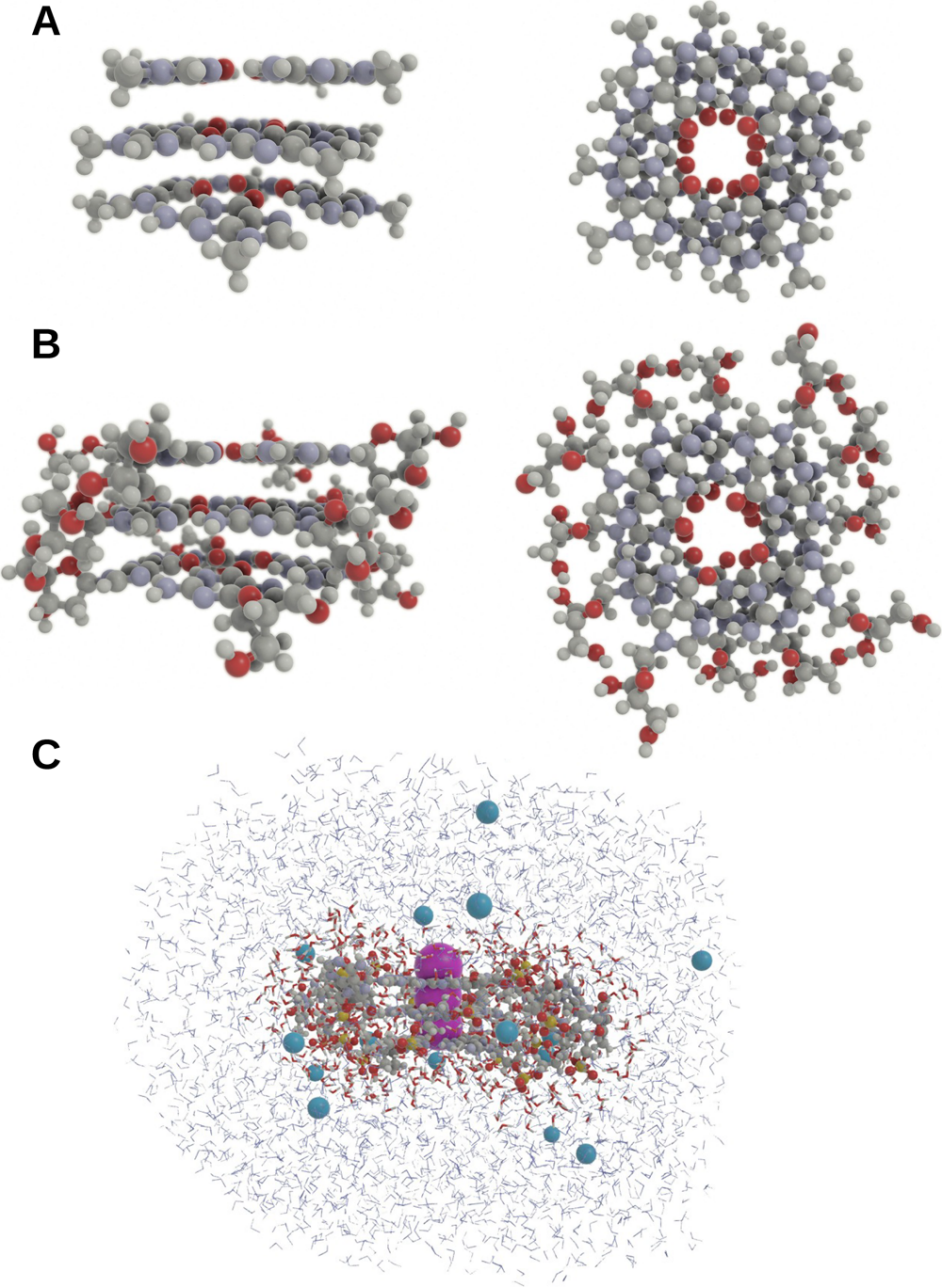
Molecular structure illustrations: (A) side and top view of the small model including guanine nucleobases; (B) side and top view of the medium model including guanine nucleosides; (C) side view of the large model including the G-quadruplex from PDB entry 1KF1. Potassium ions are represented in purple, QM water molecules are represented in the usual CPK coloring, and MM water molecules and sodium ions are represented in blue.

From the force field optimized structure obtained after an MD simulation as described in the computational details section, three model systems were built (Figure 2):

- Small: Only the 12 guanine nucleobases involved in the G-quadruplex were kept and the connections to the sugars were replaced by methyl groups. The potassium ions were not kept.
- Medium: Only the 12 guanine nucleosides involved in the G-quadruplex were kept and the connections to the phosphates were replaced by hydrogens. The potassium ions were not kept.
- Large: The full G-quadruplex was kept, including the three potassium ions. A first QM solvation layer (STO-3G) was defined as to include water and sodium within 5.5 Å from the G-quadruplex; this layer contained 565 water molecules and seven sodium ions. A second MM solvation layer was defined as to include additional water and sodium within 20 Å from the G-quadruplex; this layer contained 3788 water molecules and eight sodium ions.

### Spectrum Simulations

The strength of an electronic CD band is given by the anisotropy of the decadic molar extinction coefficient^59^
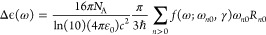
4where *N*_A_ is Avogadro’s constant, ε_0_ is the electric constant, *c* is the speed of light, *ℏ* is the reduced Planck’s constant, *f* is the Cauchy distribution, and ω_*n*0_ is the angular transition frequency between the excited state, |*n*⟩, and the ground state, |0⟩. Furthermore, *R*_*n*0_ is the rotatory strength defined as
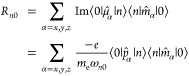
5where Im denotes the imaginary part, *e* is the elementary charge, and *m*_e_ is the electron mass. The rotatory strengths are determined in the velocity gauge as given in the second expression, and the results thereby become gauge-origin independent. The transition moments for the linear momentum, *p̂*, and magnetic dipole moment, *m̂*, operators are determined as residues of the linear response function by solving the generalized eigenvalue equation in time-dependent DFT.^59^

Alternatively, the anisotropy of the decadic molar extinction coefficient can be determined directly from the complex polarization propagator evaluated for mixed electric and magnetic dipole operators^60^
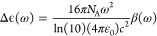
6where the molecular response property, β(ω), is defined as
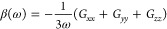
7and
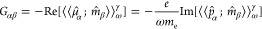
8Here, Re and Im denote the real and imaginary parts, respectively. The mixed electric–magnetic dipole tensor, *G*, is evaluated in the velocity gauge, as given in the second expression. Furthermore, it is complex and calculated with a damping term, *ℏ*γ, associated with the inverse finite lifetime of the excited states. The resulting values for Δϵ(ω) are converted from atomic units to units of L mol^–1^ cm^–1^ by multiplying with a factor of .

**Figure 3:**
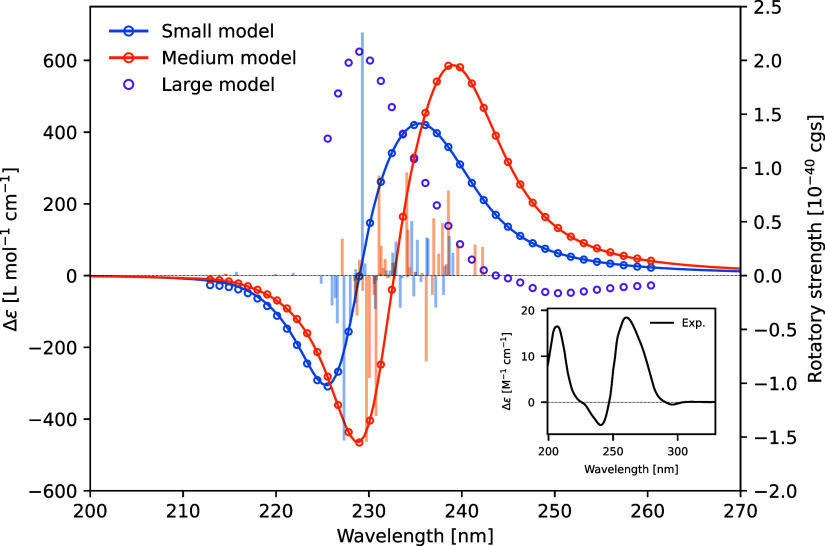
CD spectra were calculated for the three model systems. Solid lines refer to Lorentzian broadenings of the rotatory strengths depicted by bars, and circles refer to CPP results for Δϵ(ω). Inset shows the experimental spectrum reported in ref (35).

**Figure 4:**
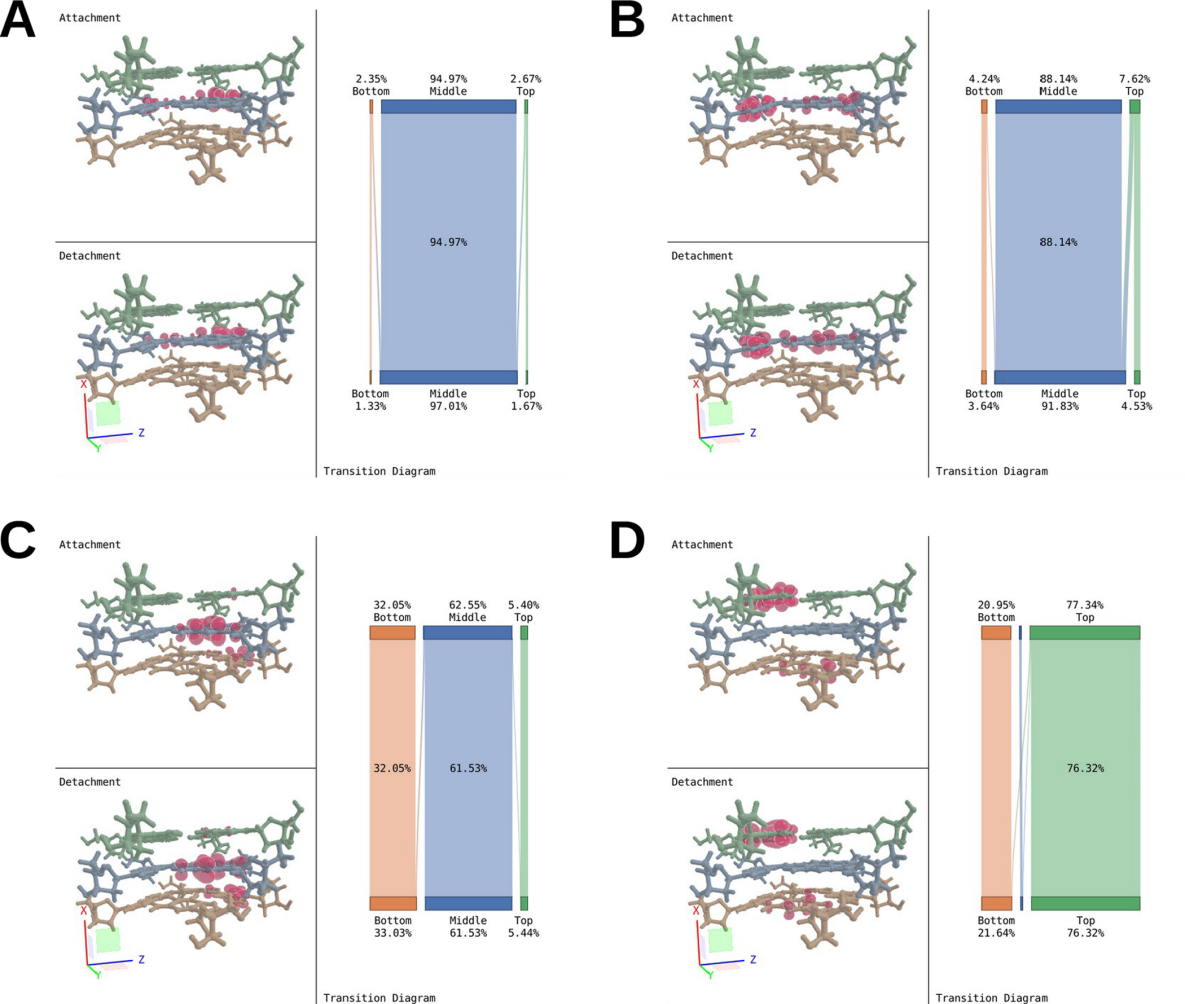
Attachment and detachment densities for the most prominent transitions for the formation of the bisignate CD band in the medium-sized model system involving nucleosides. A and B are the third and fourth transitions, respectively, contributing strongly to the positive peak, while C and D are the 27th and 30th transitions, respectively, contributing strongly to the negative peak.

In Figure 3, we plotted the CD spectra obtained for the three systems. For the small and medium model systems, spectra have been obtained both with a resolution of the 40 lowest excited singlet states (bars and solid line) as well as with the CPP method (circles). For the large system, only the CPP method was used. The three models present a clear bisignated signal characteristic of the G-quadruplex. When incorporating the sugar moiety to the G-quadruplex (from small to medium), we observe a slight red shift of 15 nm and an increase of the peak intensities but the overall shape of the spectra is not affected. To better understand the nature of the transitions at play, we focused on the medium system and looked at the attachment and detachment densities for four transitions that are shown in panels A–D in Figure 4.

We looked at these representative transitions for both the positive and negative peaks (two for each) and performed an analysis using a tool for visual analysis of electronic transitions^61^ that has been implemented in a local version of the VIAMD software.^58^ We separated the three guanine layers into separate groups, termed “bottom”, “middle”, and “top” in Figure 4, and the transition diagrams show the percentage involvement of the three groups in the electronic excitations. It is clear that these four transitions are local in character as virtually no charge transfer can be observed in between layers. Looking at the densities, it is also very clear that the excitations are predominantly localized to the guanines leaving the sugars to play a spectator role. While the two transitions contributing to the positive peak are mainly localized to the middle layer (panels A and B in Figure 4), the two transitions contributing to the negative peak have important contributions from the bottom and top layers (panels C and D in Figure 4).

**Figure 5:**
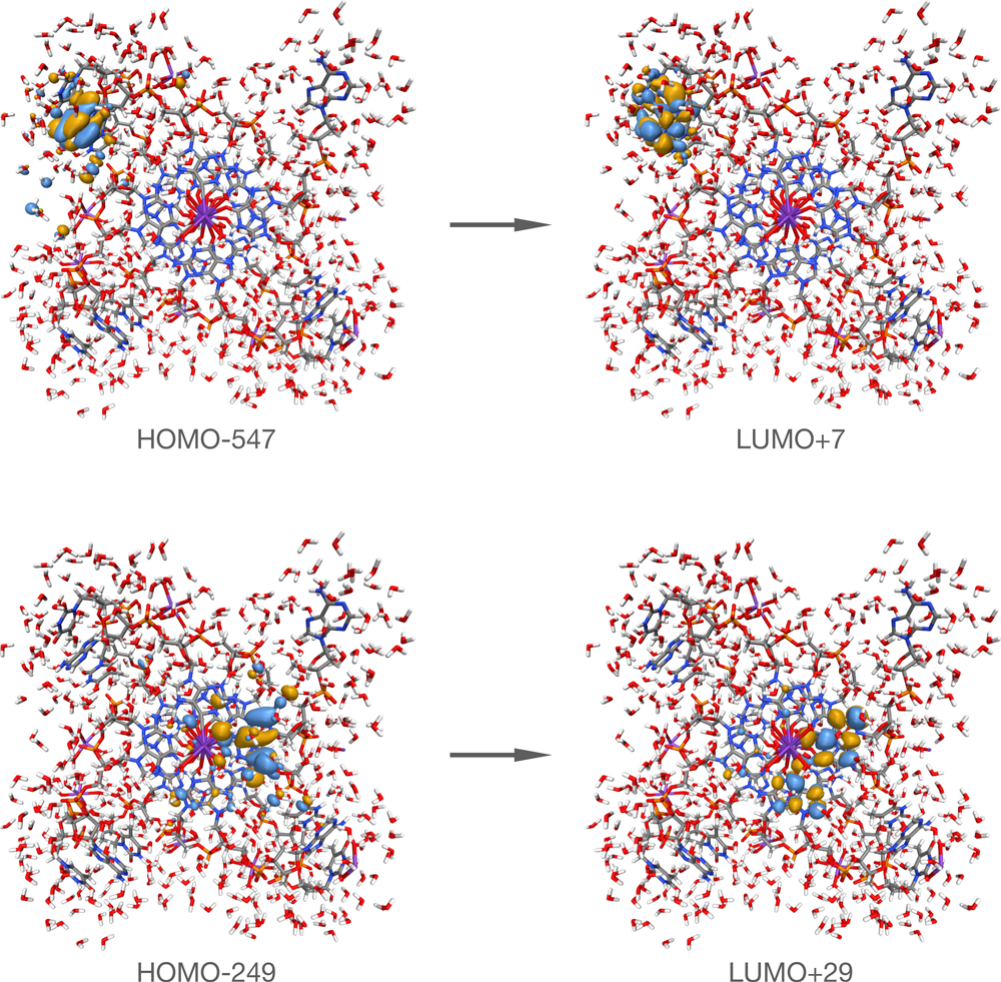
Representative excitations that contribute to the weakly negative Cotton effect at around 250 nm in the CD spectrum of the large model system.

Turning to the large model system, we observe a strong blue shift compared with the medium model system. This shift is anomalous in the sense that it opposes the trend in going from the small to the medium model system where a redshift was observed. The blue shift is also larger than the redshift such that the calculated spectrum for the large model system also becomes blue-shifted with respect to the spectrum obtained for the small model system. The overall shape of the spectrum for the large model system is largely preserved compared to the spectra for the smaller model systems. There is a small increase in the intensity of the positive band compared to the medium model system, but this effect is barely significant. More important, however, is the qualitative feature change in the long-wavelength region with respect to the small and medium models. There is here the appearance of a negative band around 250 nm that is also found present in the experiment but which has so far not been reproduced in theoretical work; we also did not find any experimental attribution for this band. In CPP calculations of Δϵ(ω), we can decompose the final result into contributions from individual orbital excitations. In general, such an analysis will show that for any value of the angular frequency, there are a large number of excitations that contribute significantly with varying positive and negative values. In the region of the negative band, however, an unexpected observation can be made, namely, the involvement of moieties other than the guanines. In Figure 5, we illustrate two excitations that both contribute with negative values to the Cotton effect. First, the excitation from occupied orbital HOMO–249 to unoccupied orbital LUMO+29, illustrated in the lower figure panel, is shown to be influenced by the potassium ions, which suggests the possible participation of the stabilizing cation in the formation of the CD spectrum. Second, the excitation is from occupied orbital HOMO–547 to unoccupied orbital LUMO+7, both of which are localized on the thymine and adenine nucleobases in the link sequence (upper figure panel). This attribution of the TTA linkers as contributors to the negative band in the long-wavelength region falls in line with an earlier study that suggested that the excitation energy of thymine is lower than that of guanine.^39^

## Conclusions

Simulations of spectroscopies for large-scale systems are challenging in several ways that energy calculations are not, at least if they are to be made practical as an analytical tool for exploring ideas and interpreting observations. In time-dependent DFT, the canonical computational task is the construction of Fock matrices, and therefore, the calculation of response properties scales identically as the SCF optimization. The key difference, however, is that in the former case, there is typically a need to determine about 2 orders of magnitude more Fock matrices as compared to the latter case. To put an emphasis on the needed responsiveness of the spectrum simulation toolbox, we have introduced a KPI in terms of the number of Fock matrices produced per hour and shown that by means of GPU acceleration, we can reach an indicator value above 26 h^–1^ for a system with close to 12,000 basis functions and with employment of a range-separated functional that has a higher computational cost for ERIs as compared to the corresponding hybrid GGA-functional.

In addition, the density of excited states is normally very high for large-scale systems, and the resolution of all the states underlying the spectral region of interest is not always feasible or at least not practical. We have demonstrated here that the adoption of the CPP formulation of response theory presents a viable way forward in this situation.

Einstein famously said:

Everything should be made as simple as possible, but not simpler.

In our context, these words of wisdom translate to the construction of suitable QM/MM models. It makes perfect sense to keep the QM region as small as possible not only for reasons of computational efforts but also because it brings focus to the studied physical phenomenon. However, it should not be made smaller than the spatial extent of the most widespread exciton of the observed spectral bands, and contrary to what previously has been claimed,^37^ the loop nucleotides do appear to have a significant impact on the CD spectra as we have been able to assign the negative Cotton effect observed experimentally, at least partially, to transitions centered on the thymine nucleotides as well as the presence of potassium ions.
